# Case report. Geschokt door de wachttijd: ESWL bij blaasstenen

**DOI:** 10.1007/s13629-021-00341-y

**Published:** 2021-10-08

**Authors:** Anna M. Zeelenberg, Nora Hendriks, Barbara M. A. Schout, Joost A. van der Spruit

**Affiliations:** afdeling Urologie, Alrijne Ziekenhuis, Leiderdorp, Nederland

**Keywords:** blaasstenen, ESWL, cystolithotripsie, COVID-19, Bladder stones, ESWL, Cystolithotripsy, COVID-19

## Abstract

De behandeling van eerste keuze voor vesicale stenen is de transurethrale cystolithotripsie (TUCL). Door de COVID-19-pandemie kampen ziekenhuizen echter met lange wachttijden voor – onder meer – deze operaties. Daarnaast komen sommige patiënten niet in aanmerking voor een operatie door hun comorbiditeit. In de jaren negentig van de vorige eeuw was extracorporele shockwave lithotripsie (ESWL) een veel gebruikte, veilige behandelmethode voor blaasstenen. Toen TUCL effectiever bleek, is ESWL voor blaasstenen echter in de vergetelheid geraakt. ESWL is poliklinisch uit te voeren, waardoor wachttijden korter zijn dan die voor operatieve ingrepen. Gedurende de COVID-19-pandemie hebben we enkele patiënten met blaasstenen behandeld met ESWL. We concluderen dat ESWL een geschikt alternatief is voor de TUCL bij geselecteerde patiënten, maar dat voor een effectieve behandeling meerdere ESWL-procedures nodig zijn.

## Abstract (en)

The first choice of treatment for vesical stones is transurethral cystolithotripsy (TUCL). However, waiting lists for surgery are long because of the COVID-19 pandemic. Additionally, some patients are not eligible for surgery due to comorbidity. During the nineties of last century, extracorporeal shockwave lithotripsy (ESWL) was a popular and safe treatment for bladder stones. When TUCL was found to be more effective, ESWL was more or less forgotten. Because ESWL can be performed on the outpatient clinic, waiting lists for ESWL are shorter than for surgical options. During the COVID-19 pandemic we have treated several patients with bladder stones with ESWL. We conclude that ESWL is a suitable alternative to TUCL for selected patients, however multiple ESWL sessions may be necessary for an effective treatment.

## Introductie

Blaasstenen zijn een vervelend en relatief veel voorkomend probleem [1, 2]. Ze vormen 5% van alle urolithiases [1]. Risicofactoren zijn onder andere bladder-outlet-obstruction zoals bij benigne prostaathyperplasie, een verblijfskatheter en neurogeen blaaslijden [2–4]. De eerste keuze bij behandeling van blaasstenen is transurethrale cystolithotripsie (TUCL) [5, 6]. In het verleden werden blaasstenen geregeld behandeld met extracorporele shock wave lithotripsie (ESWL) [7–11]. Gaandeweg kwam de TUCL steeds meer in zwang ten faveure van ESWL. Voor sommige patiënten is een TUCL echter geen optie vanwege comorbiditeit en de risico’s die anesthesie met zich meebrengt. Daarnaast is momenteel de wachttijd voor een TUCL aanzienlijk als gevolg van de druk op de ziekenhuizen door de COVID-19-pandemie.

ESWL is een minimaal invasieve behandeling die poliklinisch en zonder anesthesie kan plaatsvinden, de wachttijd is hierdoor kort. Tijdens de COVID-19-pandemie hebben we, gedreven door lange wachtlijsten, een aantal patiënten met blaasstenen behandeld met ESWL. In dit artikel presenteren we de klinische resultaten van de eerste vier op deze manier behandelde patiënten.

## Methode

De patiënten ondergingen ESWL met de Storz Modulith SLX-F2. In de periode rond de ESWL moest orale antistolling gestaakt worden, met uitzondering van acetylsalicylzuur. Van tevoren werd gecontroleerd of de blaas leeg was, zo niet, dan werd de blaas eenmalig gekatheteriseerd. Vanaf de tweede ESWL-procedure werd de blaas vooraf gespoeld om eventueel gruis te evacueren. De therapiebol werd ventraal, net craniaal van het os pubis geplaatst en stevig op de huid gedrukt om zodoende de steen in de blaas te fixeren. Voor de geleiding werd een ruime hoeveelheid gel op de therapiebol aangebracht. Per procedure werden 3.500 schokken toegediend, waarbij de energie snel werd opgehoogd tot de maximale hoogte van 30 mJ per toegediende schok. De frequentie van 2,0 schokken per seconde werd na 1.000 schokken verlaagd naar 1,5. De positie van de steen werd voor en na de procedure gecontroleerd met röntgendoorlichting. Afhankelijk van de steenload werden meerdere procedures gepland met een interval van twee weken. Alle patiënten zijn achteraf benaderd en gevraagd het effect van de behandeling te evalueren met behulp van de *Patient Global Impression of Improvement* (PGI-I)-score (tab. 1).

**Table 1:** 

“Wat beschrijft het beste uw conditie op dit moment, vergeleken met hoe het was voor de behandeling?”	
Zeer veel verbeterd	1
Veel verbeterd	2
minimaal verbeterd	3
geen verandering	4
minimaal slechter	5
veel slechter	6
zeer veel slechter	7

### Casus 1

**Figure 1:**
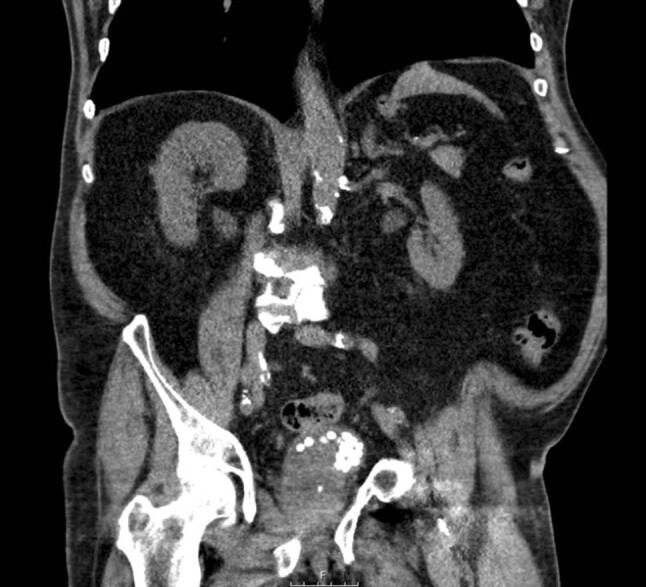

**Figure 2:**
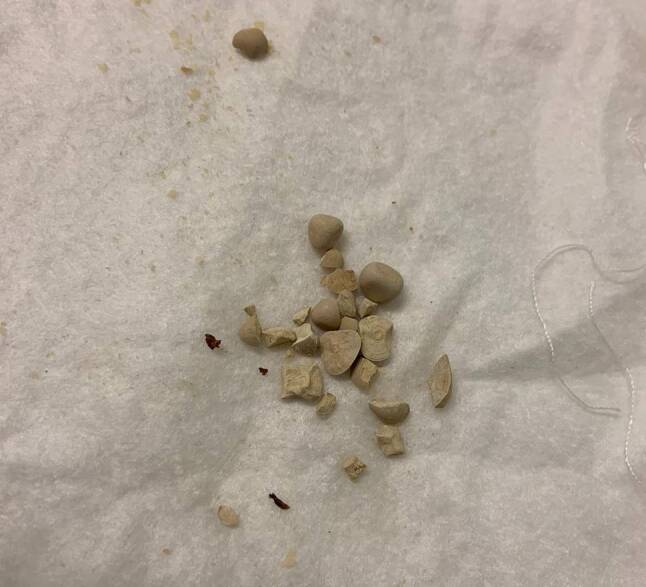

**Figure 3:**
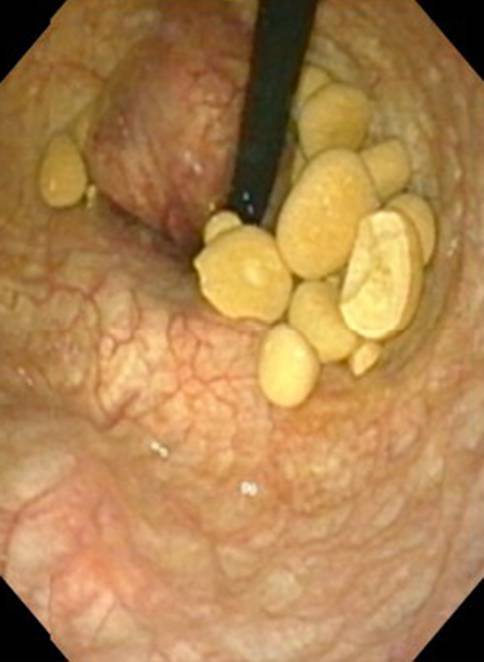

Een 83-jarige man met in de voorgeschiedenis benigne prostaathyperplasie (BPH) waarvoor dutasteride, blaasstenen van urinezuur waarvoor TUCL (2018, 2019) en alkalisering met kaliumcitraat ter metafylaxe, presenteerde zich op de polikliniek met recidiverende urineweginfecties, hematurie en strangurie. Op de blanco computertomografie (CT)-scan van de buik en bij cystoscopie werden vele stenen in de blaas gezien (fig. 1). Het aantal blaasstenen werd geschat op 45, elk met een diameter van circa 1 cm, met een gemiddelde hardheid van 500 *Hounsfield Units* (HU). Vanwege de lange wachttijd voor een TUCL werd gestart met ESWL volgens bovenstaande methode. De behandeling werd goed verdragen met een pijnscore van 1 op de *Visual Analogue Scale* (VAS). De enige complicatie was een pijnloos erytheem op de plek van de therapiebol. Na iedere procedure loosde patiënt spontaan gruis, fragmenten en soms complete stenen (fig. 2). Na de vijfde procedure werd de cystoscopie herhaald, waarop nog ongeveer een tiental stenen gezien werd (fig. 3). Bij analyse bleken het urinezuurstenen te betreffen. Inmiddels heeft patiënt de zesde procedure ook ondergaan en is hij gestart met allopurinol in het kader van metafylaxe.

Patiënt heeft geen urineweginfectie, hematurie of mictieklachten meer gehad. Het effect van de behandeling geeft hij de PGI-I-score van 1.

### Casus 2

Een 72-jarige man bekend met multiple sclerose, BPH waarvoor silodosine en blaasstenen van urinezuur waarvoor TUCL (2018, 2019) en een blaashalsresectie (2019), presenteerde zich op de polikliniek met recidiverende urineweginfecties en frequente nycturie tot zeven keer per nacht. Op een buikoverzichtsfoto werden zes blaasstenen gezien met elk een diameter van circa 1 cm; de hardheid was onbekend. Patiënt onderging ESWL volgens bovenstaande methode. Patiënt verdroeg de procedure goed met een pijnscore van 1 op de VAS. Na drie procedures vond controle plaats middels cystoscopie. Op de blaasbodem werden gruis, steenfragmenten en één nog intacte steen gezien. Na uitgebreid spoelen werden fragmenten en een grote hoeveelheid gruis verwijderd. Later passeerde patiënt spontaan twee stenen.

Patiënt heeft geen urineweginfectie of hematurie meer gehad. De nycturie is afgenomen tot drie keer per nacht, een frequentie die de patiënt acceptabel vindt. Op de buikoverzichtsfoto zijn geen blaasstenen meer zichtbaar. Steenanalyse toonde stenen van 100% urinezuur, waarna is gestart met natriumbicarbonaat ter metafylaxe. Ter evaluatie beoordeelt patiënt het effect met een PGI-I-score van 3.

### Casus 3

Een 86-jarige man bekend met prostaatcarcinoom waarvoor hormonale therapie, dementie, LUTS waarvoor alfuzosine, recidiverende urineweginfecties en blaasstenen waarvoor TUCL (2018), presenteerde zich op de polikliniek met dysurie en nycturie. Dit resulteerde in nachtelijke onrust. Bij cystoscopie werd een divertikel gezien waarin een blaassteen van circa 7 mm vast zat; de hardheid was onbekend. Patiënt onderging ESWL volgens beschreven methode en verdroeg deze goed met een pijnscore van 0 op de VAS. Tevens werd gestart met nitrofurantoïne in onderhoudsdosering. Na twee procedures werd de cystoscopie herhaald, waarop te zien was dat de steen zich niet meer in het divertikel bevond, maar in fragmenten op de bodem van de blaas lag.

Patiënt heeft geen urineweginfectie meer doorgemaakt. De nycturie is afgenomen tot één keer per nacht en de dysurie is verdwenen. De nachten zijn daardoor een stuk rustiger. De wettelijk vertegenwoordiger evalueert het effect van de behandeling met een PGI-I-score van 2.

### Casus 4

Een 55-jarige man, bekend met multipele sclerose waardoor hij rolstoelafhankelijk is en een suprapubische katheter heeft bij neurogeen blaaslijden, presenteerde zich op de polikliniek met klachten van continue aandrang en frequent lek raken van de katheterballon. Op de CT scan werd een grote blaassteen van circa 27 mm gezien. De hardheid van de steen werd geschat op 1200 HU. Vanwege zijn algehele conditie was de *American Society of Anesthesiology* (ASA)-score 4 en achtte de anesthesioloog een operatie te riskant. Aangezien er weinig curatieve opties overbleven, werd besloten om ESWL te proberen. Vanwege een spastische parese van de beide armen was het niet mogelijk om de therapiebol ventraal te plaatsen. Deze werd daarom dorsaal geplaatst. Per procedure vond vergruizing eerst plaats vanuit links van het sacrum, en daarna vanuit rechts. Hierbij werd met behulp van doorlichting op de blaassteen gericht. Per kant ontving patiënt 3.500 schokken op maximale energie. Patiënt verdroeg de behandeling goed met een pijnscore van 0 op de VAS. Na vier procedures werd de cystoscopie herhaald, waarop te zien was dat de blaassteen nog onveranderd in situ was.

De klachten zijn onverminderd aanwezig. De patiënt geeft het effect van de behandeling een PGI-I-score van 4.

## Discussie

De behandelopties voor blaasstenen variëren van blaasspoelingen, transurethrale of percutane cystolithotripsie tot de klassieke sectio alta [1, 5]. In de jaren negentig van de vorige eeuw was ESWL een gangbare behandelmethode voor blaasstenen. Studies rapporteerden succespercentages van 72–93% met één tot enkele ESWL-behandelingen [7–11]. Daarnaast werden er weinig complicaties gezien, waardoor deze behandeling veilig werd geacht [9]. Echter, toen bleek dat TUCL resulteerde in hogere steenvrije percentages, verdween ESWL naar de achtergrond. In 2019 concludeerden Donaldson et al. in een grote meta-analyse dat TUCL effectiever is dan ESWL, maar dat de studies die waren geïncludeerd van lage kwaliteit waren [5]. Inmiddels is TUCL de behandeling van eerste keus en is ESWL voor blaasstenen in de vergetelheid geraakt.

Het aantal casusbespreking in deze studie is te klein om conclusies aan te verbinden. Toch toont deze bespreking dat ESWL een optie is voor de behandeling van blaasstenen. ESWL kan poliklinisch plaatsvinden. Wel bleek de behandeling langduriger dan een operatieve ingreep. Zoals de vierde casus illustreert, is ESWL niet voor alle patiënten een geschikte optie, waarbij meerdere factoren een rol spelen, bijvoorbeeld het materiaal van de steen. In casus 1 en 2 betrof het urinezuurstenen, die zich gemakkelijk laten vergruizen; bij een zeer harde of grote steen, zoals in casus 4, is vergruizen moeilijker [10, 11]. Verder speelt het kunnen positioneren van de therapiebol een rol. In casus 4 kon de therapiebol niet in optimale positie worden gebracht, omdat vergruizen vanaf dorsaal, wat bij deze patiënt een logische positie was, wordt gehinderd door de ossale structuur van het sacrum. Dan de beweeglijkheid van de steen. Voor een effectieve ESWL moet de steen constant in dezelfde positie blijven, maar een steen in de blaas is redelijk mobiel. De steen kan wel zo goed mogelijk worden gefixeerd door de blaas voor de behandeling te ledigen en de therapiebol stevig op de onderbuik te laten duwen. Dan de zichtbaarheid van de stenen. Hoewel blaasstenen niet altijd zichtbaar zijn met doorlichting, hoeft dit geen probleem te zijn, dankzij de bekende locatie van de stenen. Is zichtbaarheid wel gewenst, dan is echografiegeleide ESWL een optie.

Bij alle patiënten waren meerdere ESWL-behandelingen nodig voor het bereiken van adequate vergruizing. Bij een obstruerende prostaat of bij een verblijfskatheter blijft na vergruizing regelmatig gruis achter, waarop weer nieuwe steenaanslag kan ontstaan. Daarom is het in die gevallen vaak noodzakelijk om het overgebleven gruis te evacueren middels spoeling van de blaas.

## Conclusie

Hoewel ESWL inferieur is aan TUCL, is het een non-invasieve en veilige therapie waarmee blaasstenen adequaat behandeld kunnen worden. Wanneer de operatieve mogelijkheden beperkt zijn, bijvoorbeeld in het geval van lange wachttijden of vanwege contra-indicaties, is ESWL een geschikt alternatief om een geselecteerde patiëntengroep te ontdoen van hun blaasste(n)en en de bijkomende klachten. Patiënten moeten erover geïnformeerd worden dat meestal meerdere ESWL- procedures nodig zijn en dat vaak het achtergebleven gruis uitgespoeld moet worden.
